# Supplementary material for: Synthesis of New Tricyclic 1,2-Thiazine Derivatives with Anti-Inflammatory Activity
Source: Int J Mol Sci. 2021 Jul 22;22(15):7818. doi: 10.3390/ijms22157818 (PMC8346139; doi:10.3390/ijms22157818)
Supplement: Supplementary file 1 [file ijms-22-07818-s001.zip › ijms-1275670-supplementary.pdf]

Supplementary materials

Synthesis of New Tricyclic 1,2-Thiazine Derivatives with Anti-Inflammatory Activity

Jadwiga Maniewska [1], Benita Wiatrak [2], Żaneta Czyżnikowska [3] and Berenika M. Szczęśniak-Sięga [1]

1 Department of Medicinal Chemistry, Faculty of Pharmacy, Wroclaw Medical University, Borowska 211, 50-556 Wrocław, Poland
2 Department of Pharmacology, Faculty of Medicine, Wroclaw Medical University, J. Mikulicza-Radeckiego 2, 50-345 Wrocław, Poland
3 Department of Inorganic Chemistry, Faculty of Pharmacy, Wroclaw Medical University, Borowska 211a, 50-556 Wrocław, Poland

^{1}H NMR and ^{13}C NMR spectra

compound	structure	page	compound	structure	page
**5**	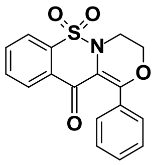	S2	**6d**	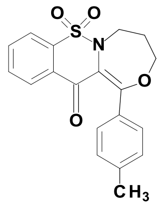	S6
**6a**	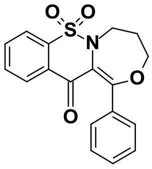	S3	**6e**	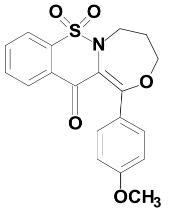	S7
**6b**	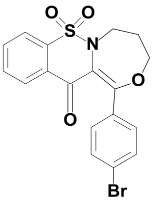	S4	**7**	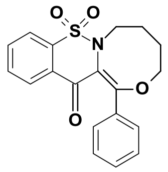	S8
**6c**	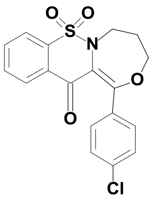	S5			

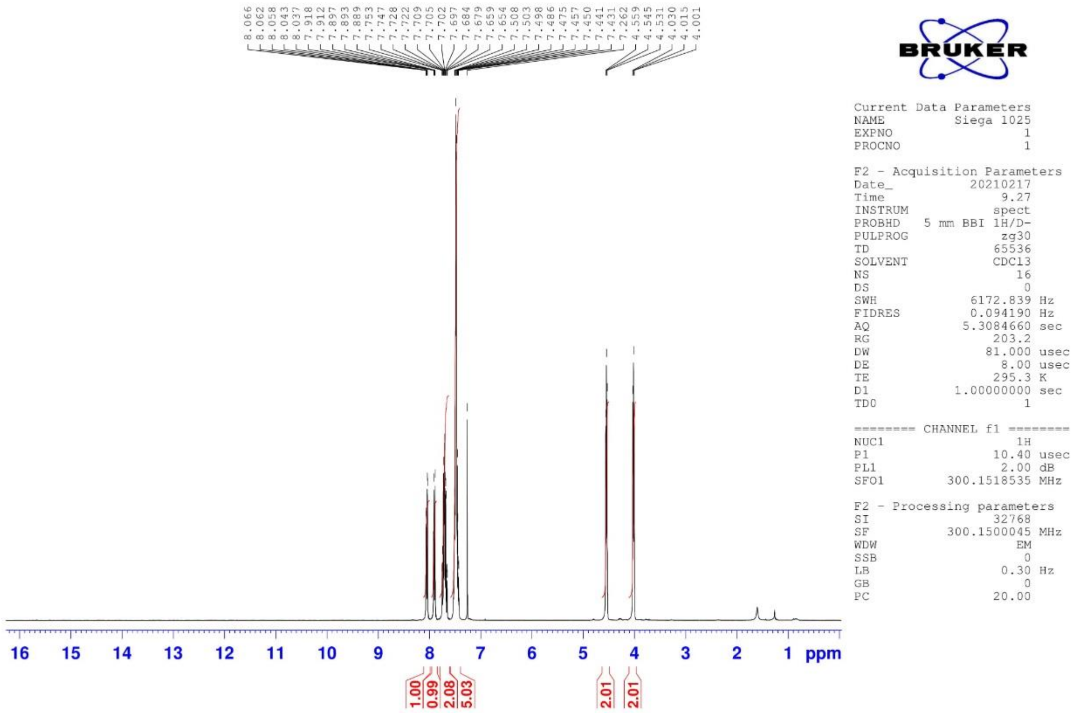

Figure S1. ^{1}H NMR spectrum of **5** in $CDCl_3$.

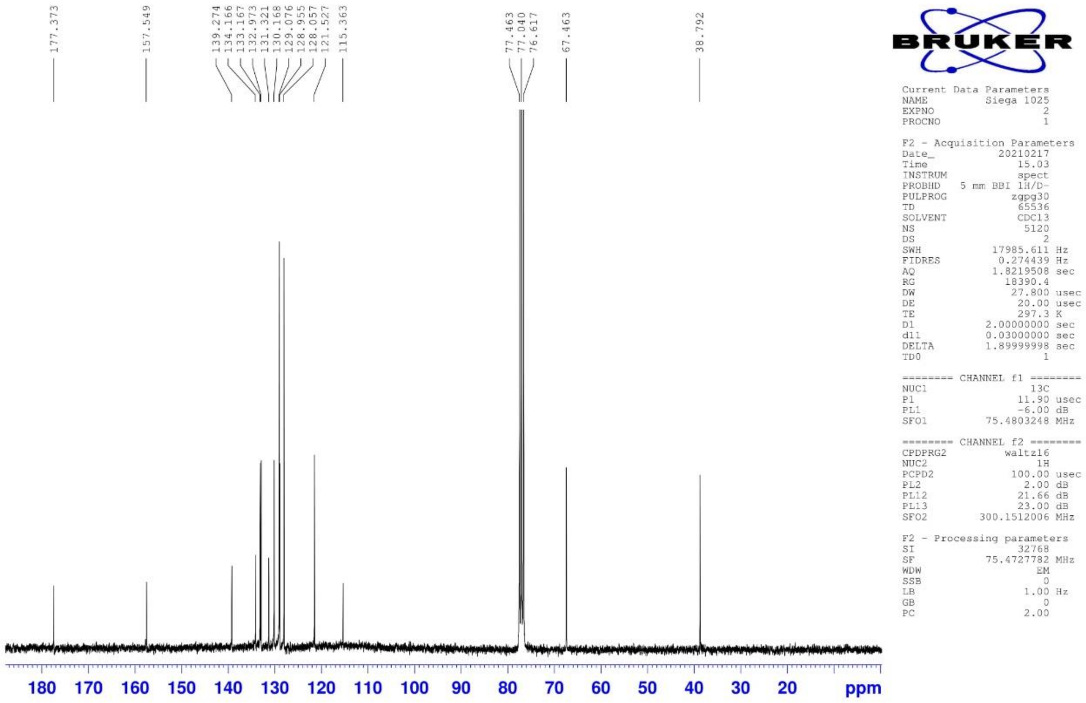

Figure S2. ^{13}C NMR spectrum of **5** in $CDCl_3$.

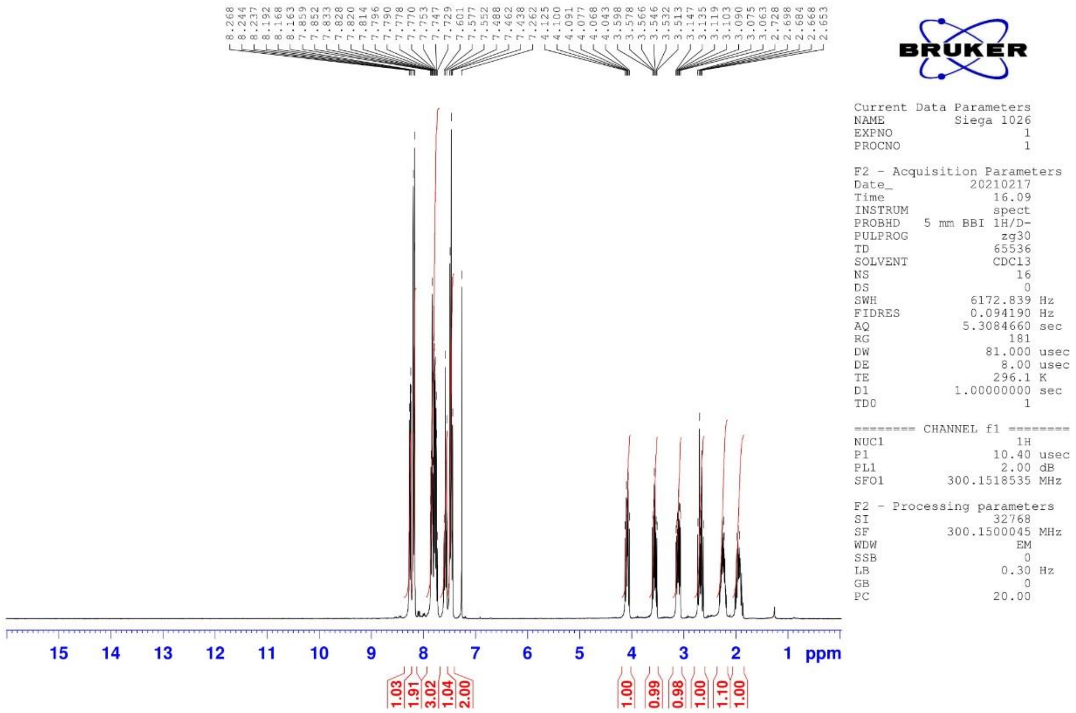

Figure S3. ^{1}H NMR spectrum of **6a** in $CDCl_3$.

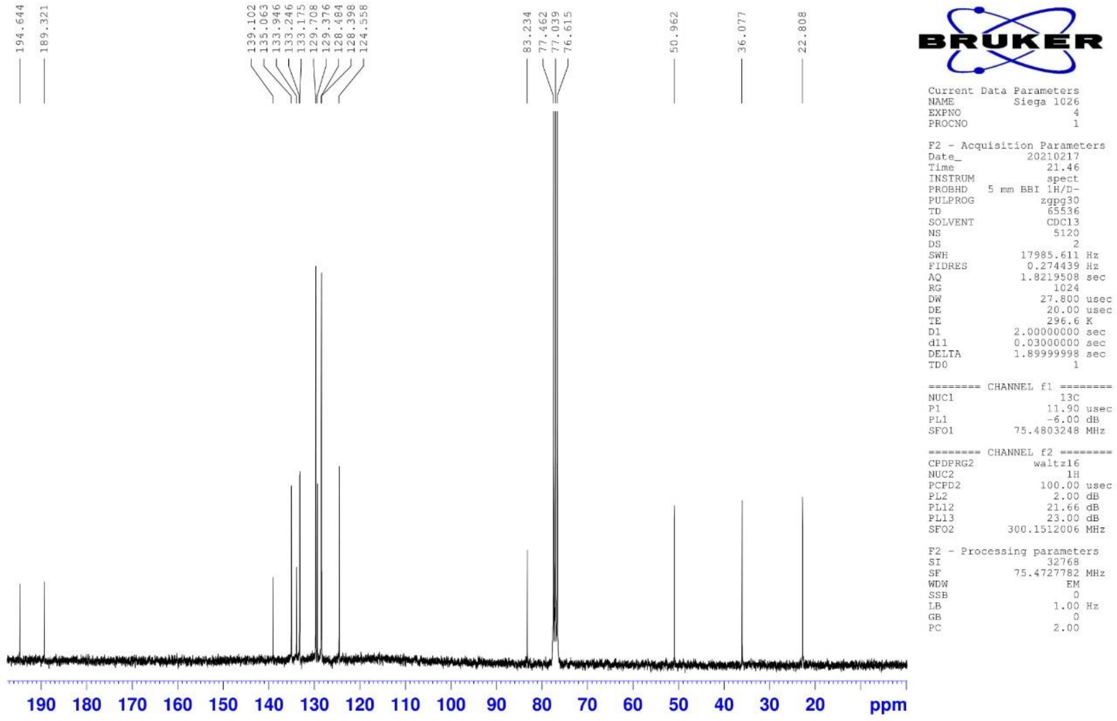

Figure S4. ^{13}C NMR spectrum of **6a** in $CDCl_3$.

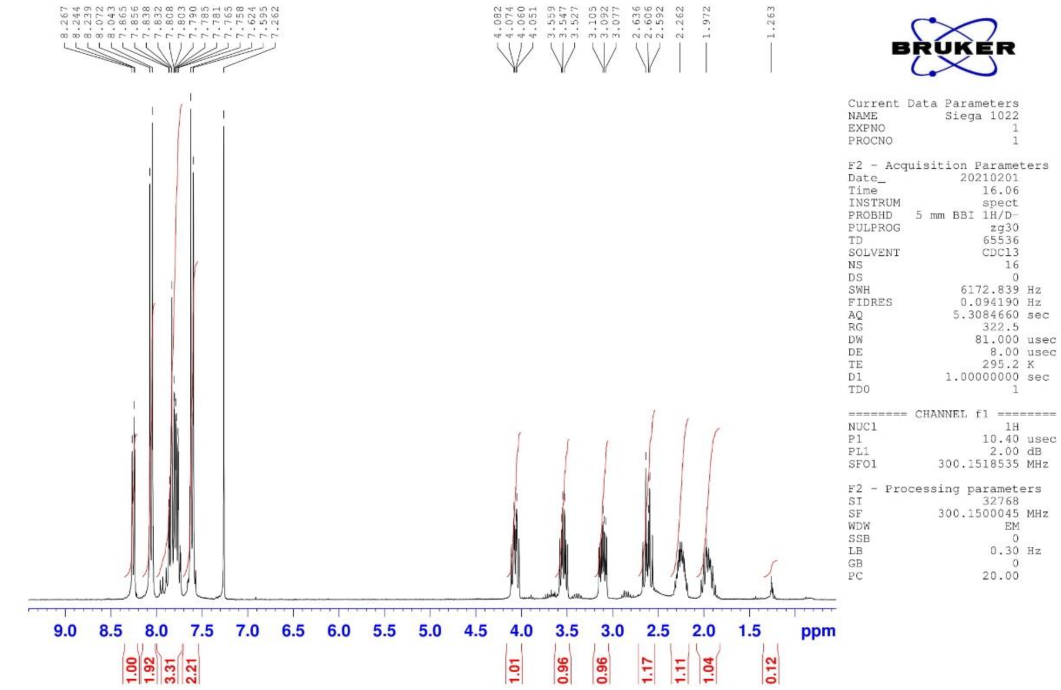

Figure S5. ^{1}H NMR spectrum of **6b** in $CDCl_3$.

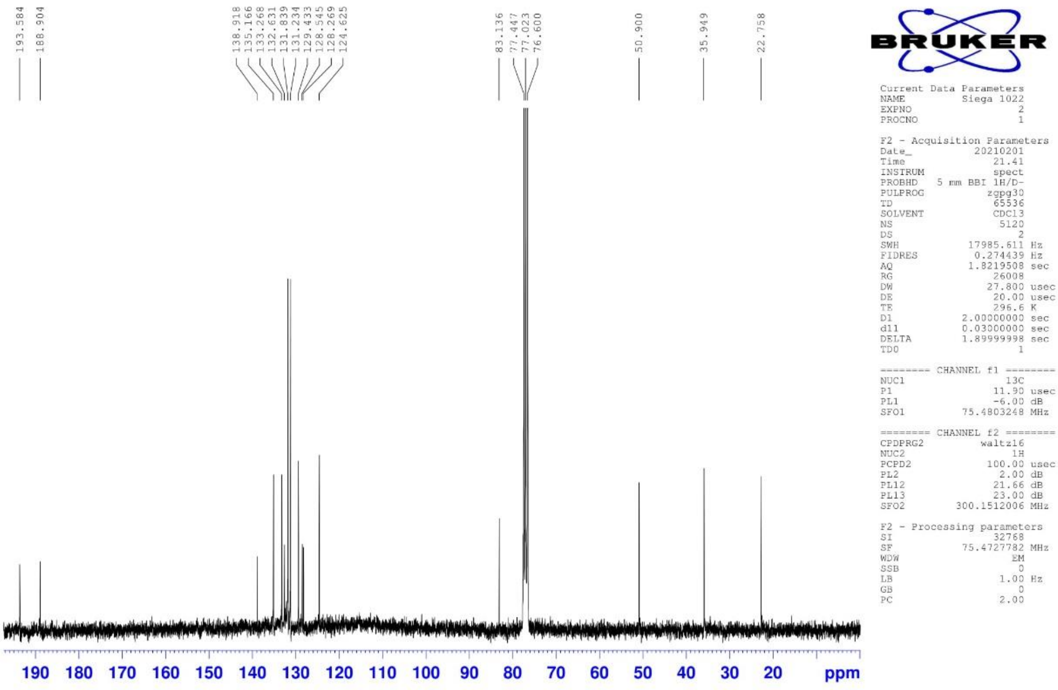

Figure S6. ^{13}C NMR spectrum of **6b** in $CDCl_3$.

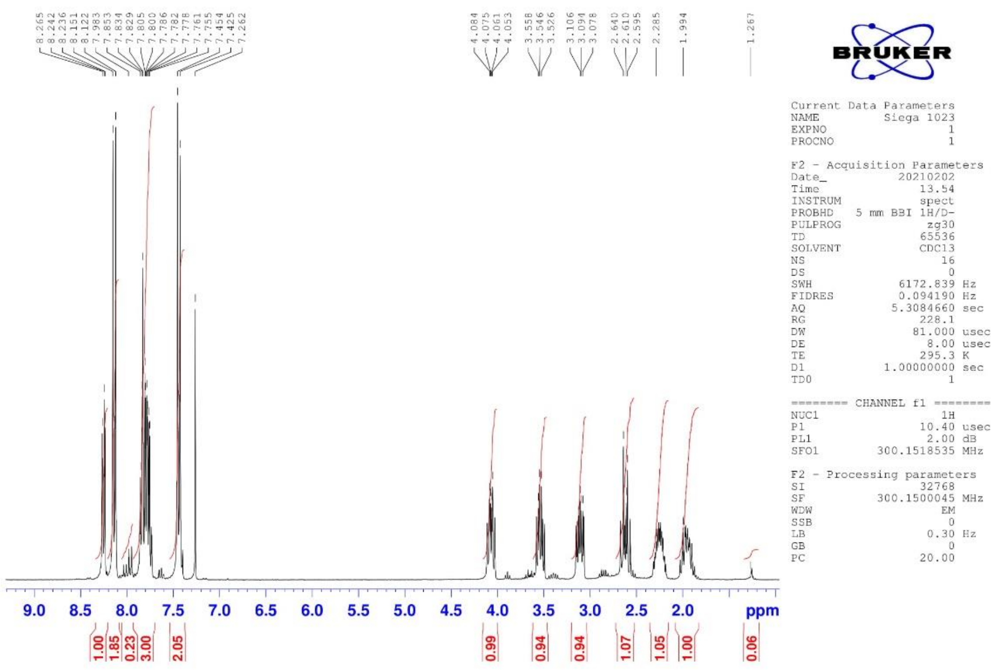

Figure S7. ^{1}H NMR spectrum of **6c** in $CDCl_3$.

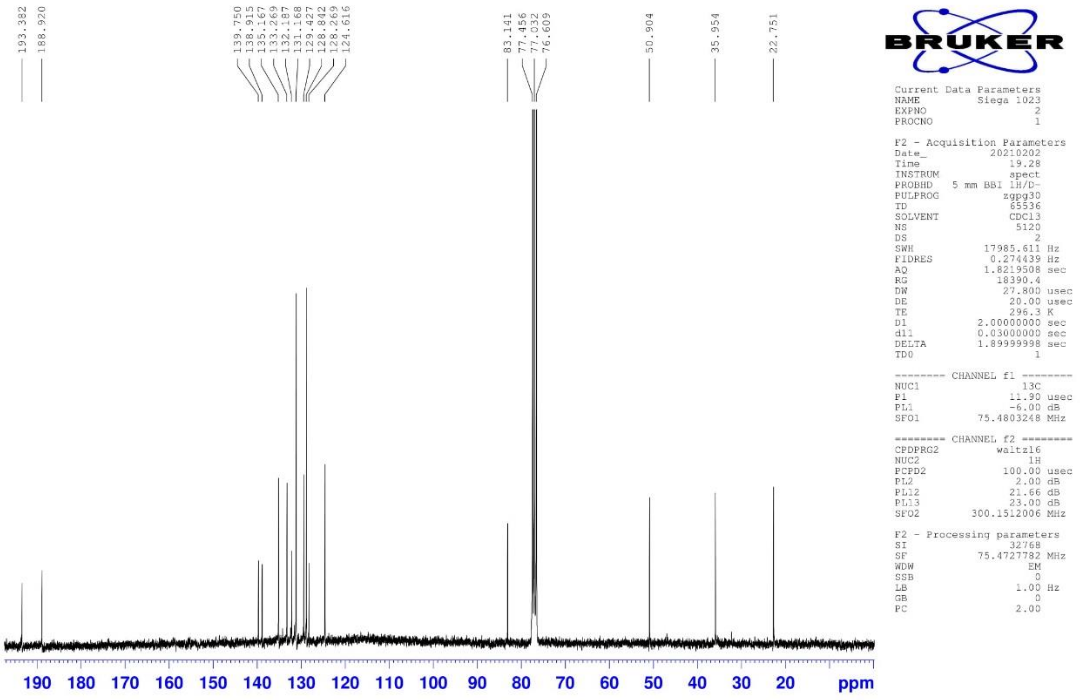

Figure S8. ^{13}C NMR spectrum of **6c** in $CDCl_3$.

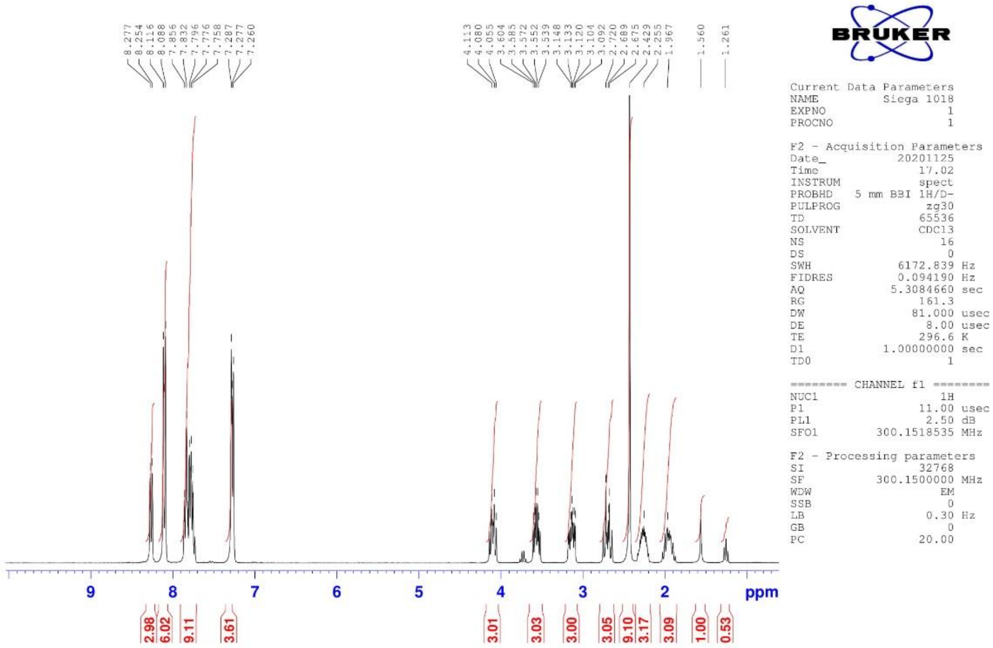

Figure S9. ^{1}H NMR spectrum of **6d** in $CDCl_3$.

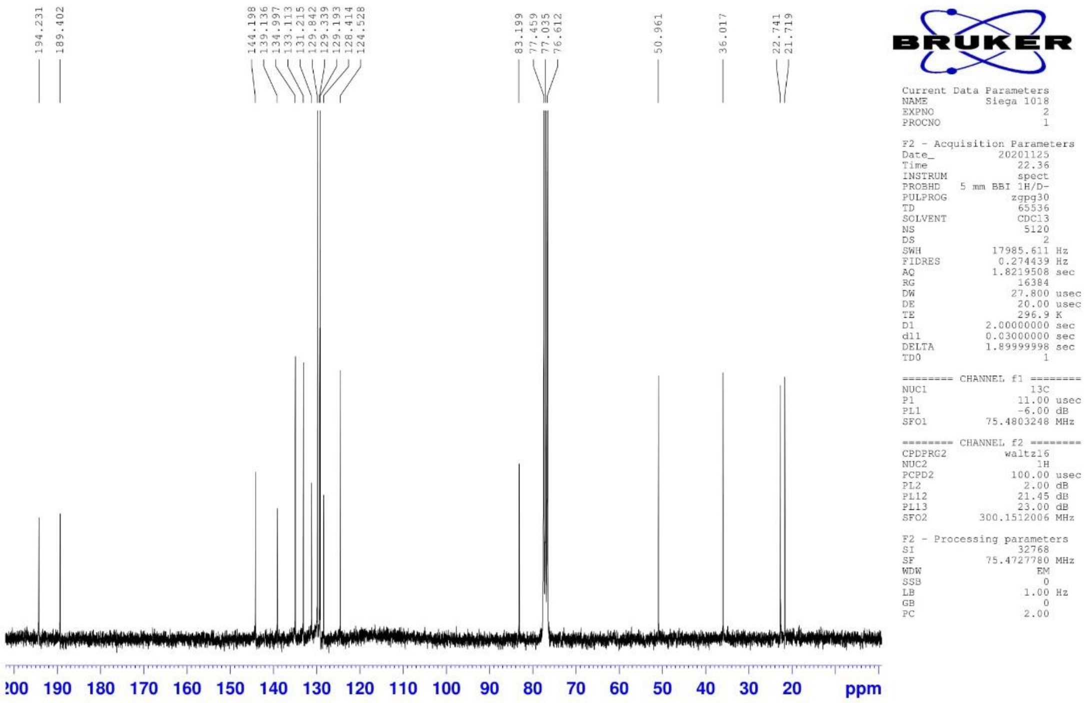

Figure S10. ^{13}C NMR spectrum of **6d** in $CDCl_3$.

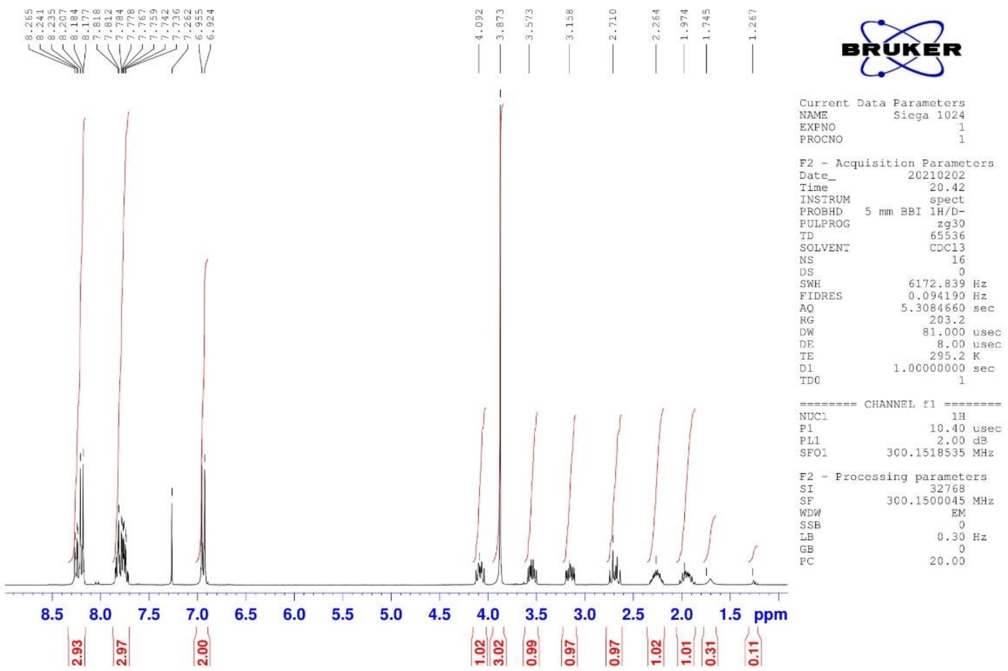

Figure S11. ^{1}H NMR spectrum of **6e** in $CDCl_3$.

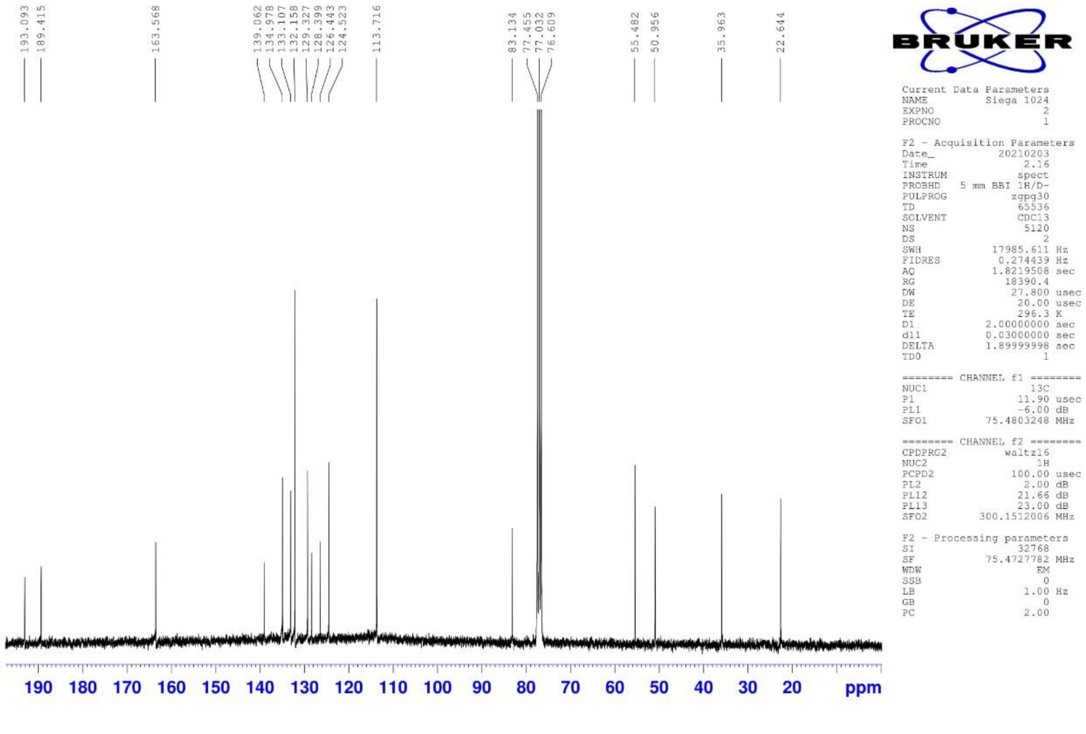

Figure S12. ^{13}C NMR spectrum of **6e** in $CDCl_3$.

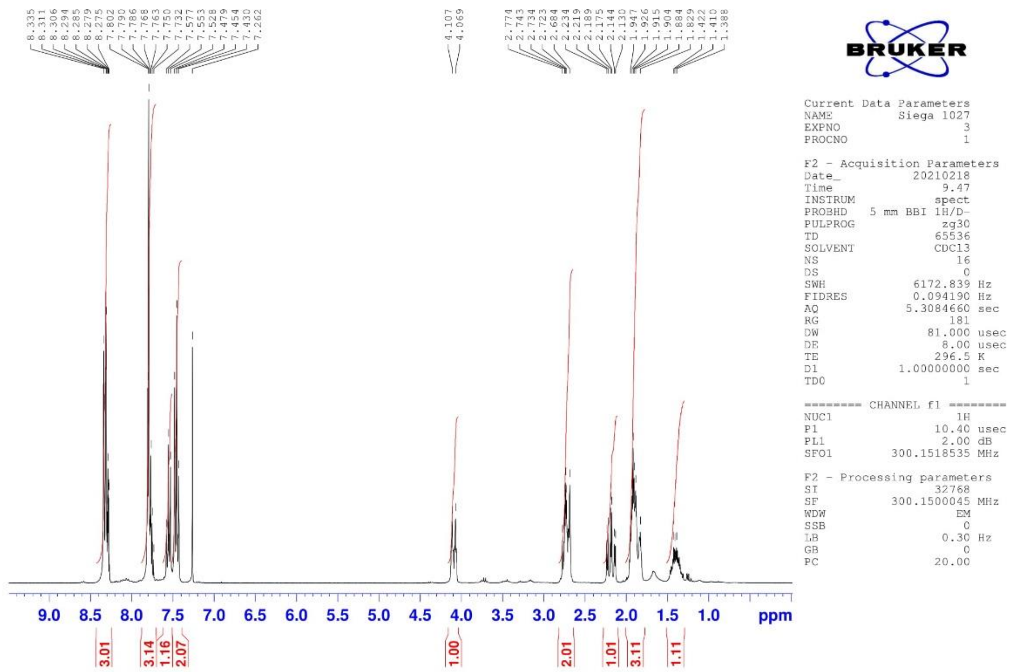

Figure S13. ^{1}H NMR spectrum of **7** in $CDCl_3$.

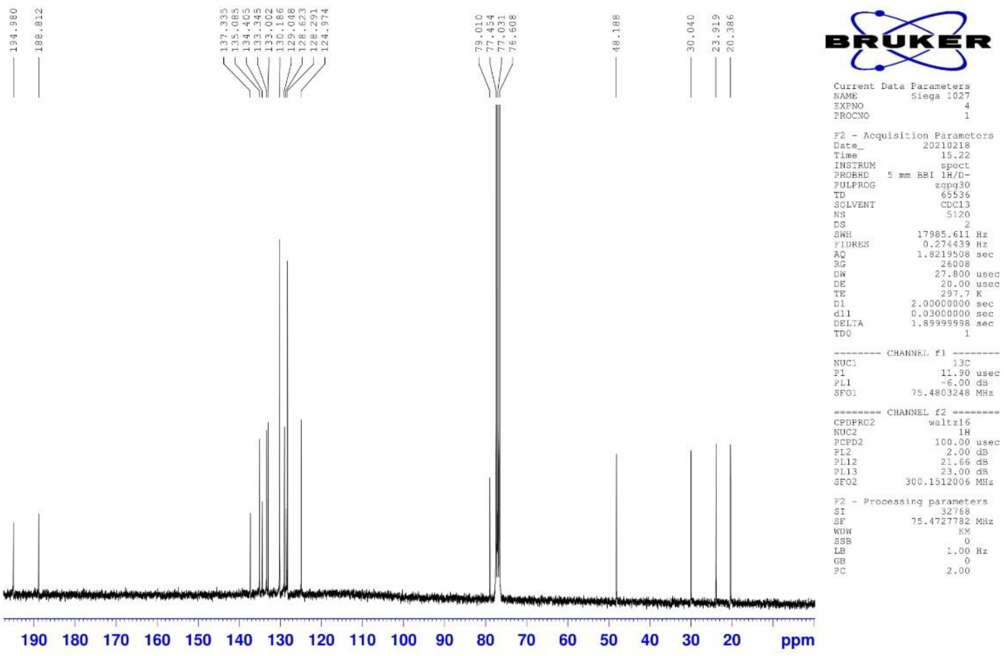

Figure S14. ^{13}C NMR spectrum of **7** in $CDCl_3$.

The influence of new 1,2-thiazine derivatives on the thermotropic properties of DPPC model membranes

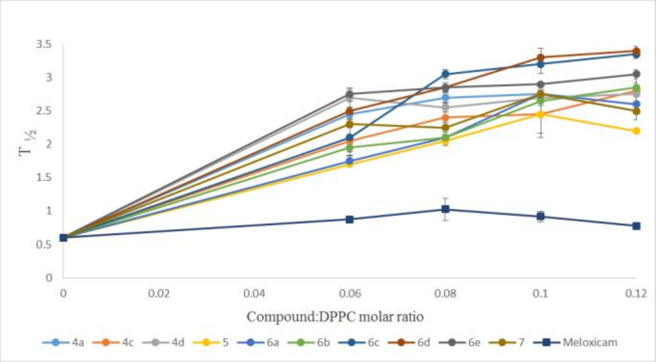

Figure S15. The effect of 1,2-thiazine derivatives in comparison to the meloxicam on the transition half height ($T_{1/2}$) of the main phase transition of DPPC.

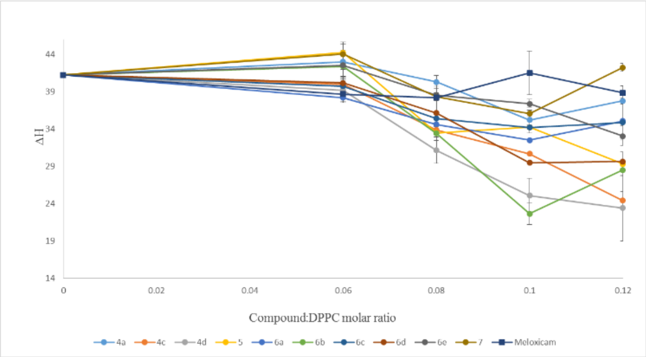

Figure S16. The effect of 1,2-thiazine derivatives in comparison to the meloxicam on the transition enthalpy (ΔH) of the main phase transition of DPPC

Table S1. Values of DPPC phase transition temperatures (T_M) with studied compounds

Compound	DPPC T_M values for different molar ratios of compound studied:lipid ±SD			
	0.06	0.08	0.1	0.12
4a	38,85 ± 0.07	38,4 ± 0.14	37,9 ± 0.14	37,85 ± 0.07
4c	39,35 ± 0.09	39,05 ± 0.07	39,1 ± 0.03	38,75 ± 0.21
4d	39,95 ± 0.04	38,5 ± 0.02	38,05 ± 0.07	38,35± 0.20
5	40,15 ± 0.07	39,75 ± 0.08	40 ± 0.02	39,35 ± 0.06
6a	39,95 ± 0.07	39,55 ± 0.07	38,7 ± 0.14	38,65 ± 0.07
6b	40,05 ± 0.08	40,6 ± 0.02	40 ± 0.14	39,25 ± 0.06
6c	39,2 ± 0.06	38,5 ± 0.06	37,6 ± 0.02	37,1 ± 0.13
6d	39 ± 0.00	38,5 ± 0.00	37,25 ± 0.08	37,15 ± 0.06
6e	39,2 ± 0.14	38,9 ± 0.02	38,65 ± 0.07	38,2 ± 0.03
7	39,1 ± 0.06	39,1 ± 0.03	38,5 ± 0.03	38,9 ± 0.13
meloxicam	40,62 ± 0.05	40,69 ± 0.03	40,72 ± 0.04	40,7 ± 0.00

Table S2. Values of DPPC transition half height ($T_{½}$) with studied compounds

Compound	DPPC $T_{½}$ values for different molar ratios of compound studied:lipid ±SD			
	0.06	0.08	0.1	0.12
4a	2.45 ± 0.07	2.7 ± 0.07	2.75 ± 0.07	2.5 ± 0.13
4c	2.05 ± 0.21	2.4 ± 0.14	2.45 ± 0.28	2.8 ± 0.04
4d	2.7 ± 0.13	2.55 ± 0.05	2.7 ± 0.07	2.75 ± 0.28
5	1.7 ± 0.03	2.05 ± 0.07	2.45 ± 0.35	2.2 ± 0.04
6a	1.75 ± 0.06	2.1 ± 0.00	2.75 ± 0.07	2.6 ± 0.14
6b	1.95 ± 0.07	2.1 ± 0.03	2.65 ± 0.07	2.85 ± 0.07
6c	2.1 ± 0.14	3.05 ± 0.07	3.2 ± 0.14	3.35 ± 0.07
6d	2.5 ± 0.00	2.85 ± 0.07	3.3 ± 0.02	3.4 ± 0.00
6e	2.75 ± 0.06	2.85 ± 0.05	2.9 ± 0.00	3.05± 0.07
7	2.3 ± 0.03	2.25± 0.07	2.75 ± 0.07	2.5 ± 0.07
meloxicam	0.87 ± 0.05	1.02 ± 0.17	0.92± 0.07	0.77 ± 0.05

Table S3. Values of DPPC transition enthalpy changes (ΔH) with studied compounds

Compound	DPPC ΔH values for different molar ratios of compound studied:lipid ±SD			
	0.06	0.08	0.1	0.12
4a	42.9 ± 2.29	40.3 ± 0.85	35.2 ± 0.99	37.8 ± 0.43
4c	40.0 ± 1.94	33.9 ± 1.41	30.7 ± 0.11	24.4 ± 1.16
4d	39.2 ± 1.71	31.2 ± 1.78	25.1 ± 2.27	23.4 ± 4.41
5	44.2 ± 1.43	33.5 ± 0.09	34.2 ± 0.79	29.3 ± 1.61
6a	38.2 ± 0.20	34.6 ± 0.27	32.5 ± 0.12	35.1 ± 0.18
6b	42.4 ± 0.06	33.4 ± 0.52	22.6 ± 1.43	28.5 ± 0.81
6c	39.6 ± 0.81	35.4 ± 0.01	34.2 ± 0.29	34.9 ± 3.09
6d	40.2 ± 0.12	36.1 ± 0.60	29.5 ± 0.19	29.6 ± 0.31
6e	42.5 ± 1.51	38.5 ± 0.53	37.4 ± 0.43	33.0 ± 0.51
7	44.0 ± 2.06	38.3 ± 1.17	36.1 ± 0.64	42.2 ± 1.43
meloxicam	38.7 ± 0.93	38.2 ± 2.88	41.5 ± 2.91	38.8 ± 0.93

Molecular docking studies

4a - COX-1

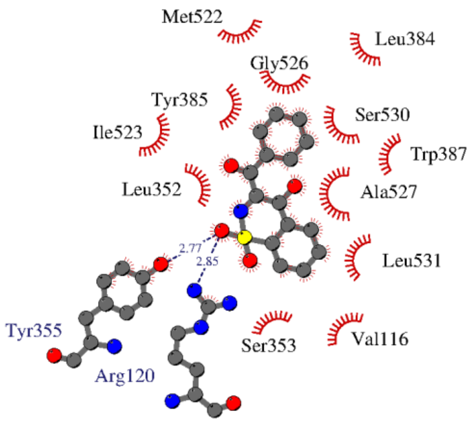

4a - COX-2

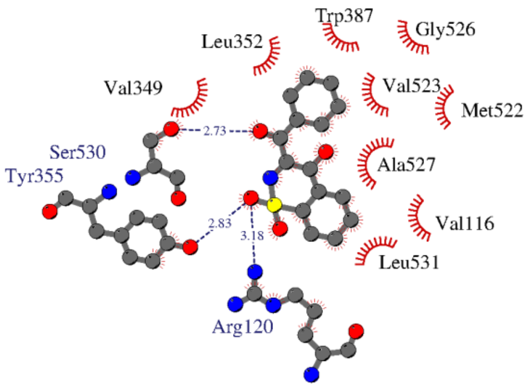

4c - COX-1

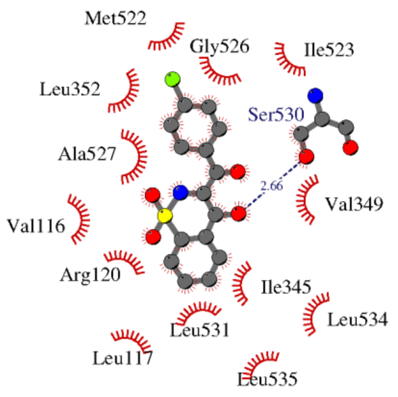

4c - COX-2

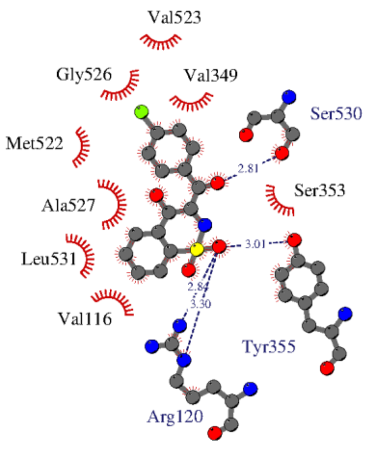

4d - COX-1

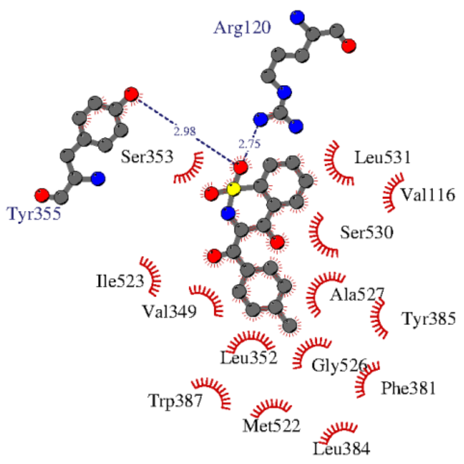

4d - COX-2

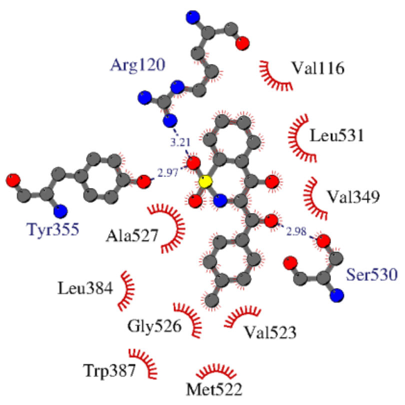

5 - COX-1

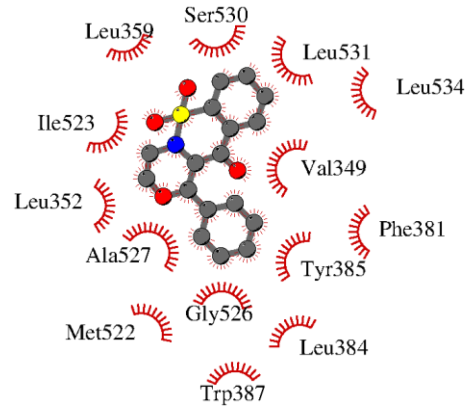

5 - COX-2

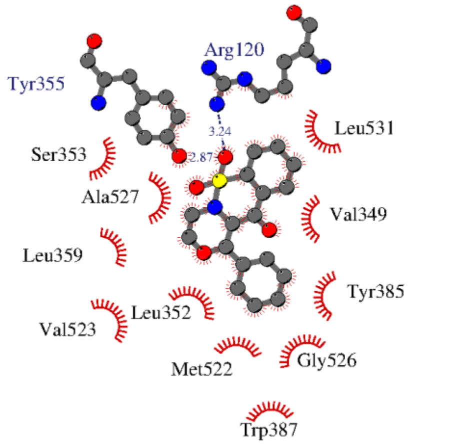

6a - COX-1

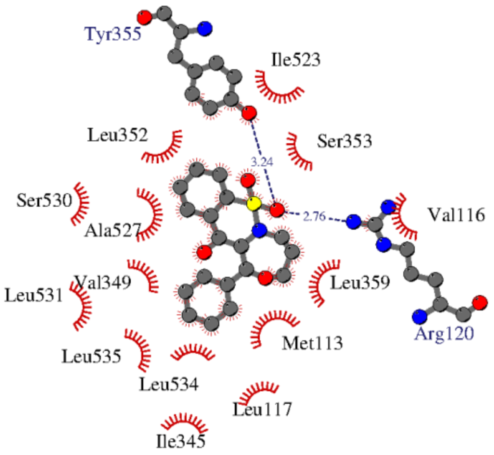

6a - COX-2

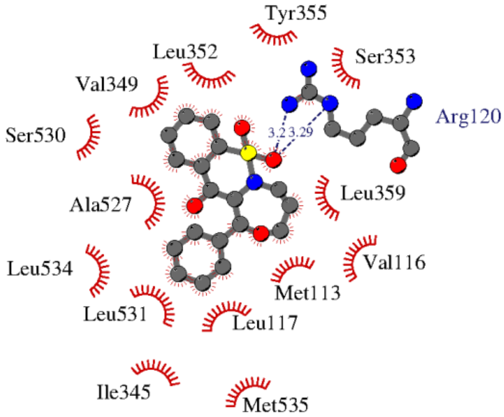

6b - COX-1

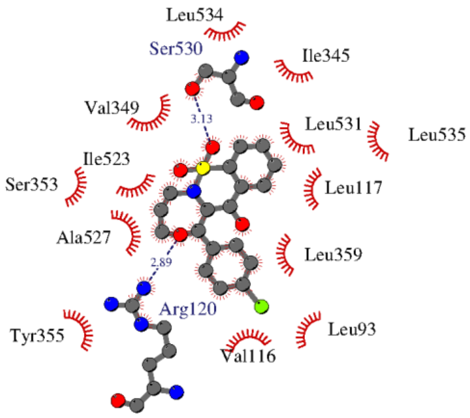

6b - COX-2

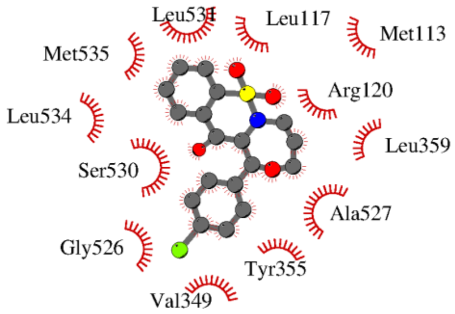

6c - COX-1

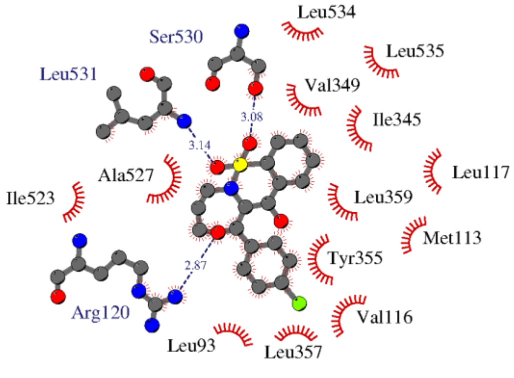

6c - COX-2

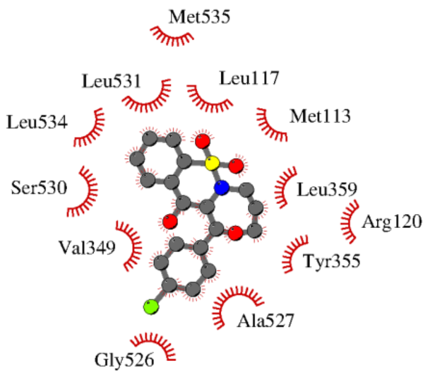

6d - COX-1

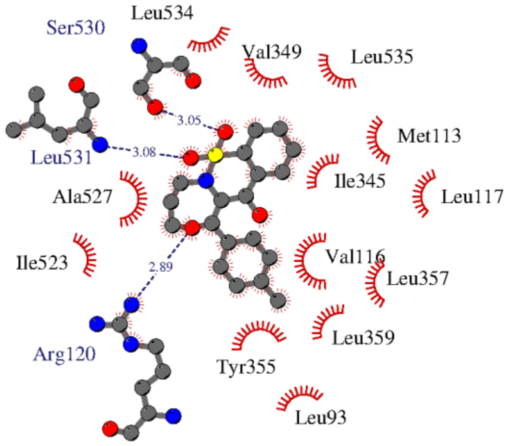

6d - COX-2

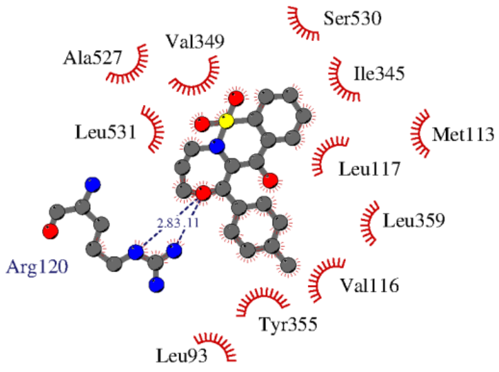

7 - COX-1

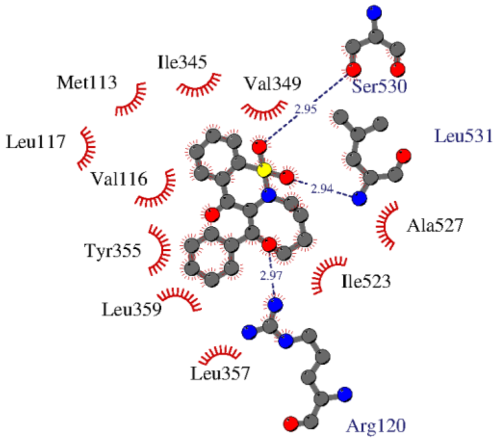

7 - COX-2

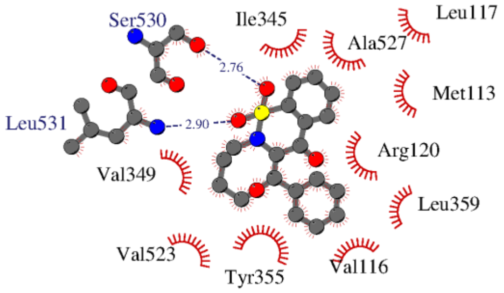

Figure S17. The intermolecular interactions between COX-1 and COX-2 and designed compounds (hydrophobic as red arches and hydrogen bonds as navy blue lines).

Table S4. Free energy of binding and intermolecular interactions of new 1,2-benzothiazine derivatives with COX-1.

Compound	Free energy of binding to COX-1 [kcal/mol]	Hydrogen bonds (distance between electronegative atoms)	Hydrophobic interactions
**4a**	-8.9	Arg120 (2.85 Å)	Val116, Arg120, Leu352, Ser353, Tyr355, Leu384, Tyr385, Trp387, Met522, Ile523, Gly526, Ala527, Ser530, Leu531
**4c**	-10.0	Tyr355 (2.77 Å)	Val116, Leu117, Arg120, Ile345, Val349, Leu352, Met522, Ile523, Gly526, Ala527, Ser530, Leu531, Leu534, Leu535
**4d**	-9.0	Ser530 (2.66 Å)	Val116, Arg120, Val349, Leu352, Ser353, Tyr355, Phe381, Leu384, Tyr385, Trp387, Met522, Ile523, Gly526, Ala527, Ser530, Leu531
**5**	-9.7	Arg120 (2.75 Å)	Val349, Leu352, Leu359, Phe381, Leu384,Tyr385, Trp387, Met522, Ile523, Gly526, Ala527, Ser530, Leu531, Leu534
**6a**	-9.4	Tyr355 (2.98 Å)	Met113, Val116, Leu117, Arg120, Leu345, Val349, Leu352, Ser353, Tyr355, Leu359, Ile523, Ala527, Ser530, Leu531, Leu534, Leu535
**6b**	-9.7	–	Leu93, Val116, Leu117, Arg120, Ile345, Val349, Ser353,Tyr355, Leu359, Ile523, Ala527, Ser530, Leu531, Leu534, Leu535
**6c**	-9.5	Arg120 (2.76 Å)	Leu93, Met113, Val116, Leu117, Arg120, Ile345, Val349, Tyr355, Leu357, Leu359, Ile523, Ala527, Ser530, Leu531, Leu534, Leu535
**6d**	-9.3	Tyr355 (3.24 Å)	Leu93, Met113, Val116, Leu117, Arg120, Ile345, Val349, Tyr355, Leu357, Leu359, Ile523, Ala527, Ser530, Leu531, Leu534, Leu535
**6e**	-8.9	Arg120 (2.89 Å)	Leu93, Met113, Val116, Leu117, Arg120, Ile345, Val349, Tyr355, Leu357, Leu359, Ile523, Ala527, Ser530, Leu531, Leu534, Leu535
**7**	-8.7	Ser530 (3.13 Å)	Met113, Val116, Leu117, Arg120, Ile345, Val349, Tyr355, Leu357, Leu359, Ala527, Ser530, Leu531

Table S5. Free energy of binding and intermolecular interactions of new 1,2-benzothiazine derivatives with COX-2.

Compound	Free energy of binding to COX-2 [kcal/mol]	Hydrogen bonds (distance between electronegative atoms)	Hydrophobic interactions
**4a**	-11.2	Arg120 (3.18 Å)<br>Tyr355 (2.83 Å)<br>Ser530 (2.73 Å)	Val116, Arg120, Val349, Leu352, Tyr355, Trp387, Met522, Val523, Gly526, Ala527, Ser530, Leu531
**4c**	-12.6	Arg120 (2.84 Å, 3.30 Å)<br>Tyr355 (3.01 Å)<br>Ser530 (2.81 Å)	Val116, Arg120, Val349, Ser353, Tyr355, Met522, Val523, Gly526, Ala527, Ser530, Leu531
**4d**	-9.4	Arg120 (3.21 Å)<br>Tyr355 (2.97 Å)<br>Ser530 (2.98 Å)	Val116, Arg120, Val349, Tyr355, Leu384, Trp387, Met522, Val523, Gly526, Ala527, Ser530, Leu531
**5**	-9.8	Arg120 (3.24 Å)<br>Tyr355 (2.87 Å)	Arg120, Val349, Leu352, Ser353, Tyr355, Leu359, Tyr385, Trp387, Met522, Val523, Gly526, Ala527, Leu531
**6a**	-9.5	Arg120 (3.23 Å, 3.29 Å)	Met113, Val116, Leu117, Arg120, Ile345, Val349, Leu352, Ser353, Tyr355, Leu359, Ala527, Ser530, Leu531, Leu534, Met535
**6b**	-8.8	–	Met113, Leu117, Arg120, Val349, Tyr355, Leu359, Gly526, Ala527, Ser530, Leu531, Leu534, Met535
**6c**	-9.2	–	Met113, Leu117, Arg120, Val349, Tyr355, Leu359, Gly526, Ala527, Ser530, Leu531, Leu534, Met535
**6d**	-9.4	Arg120 (2.83 Å, 3.11 Å)	Leu93, Met113, Val116, Leu117, Arg120, Ile345, Val349, Tyr355, Leu359, Ala527, Ser530, Leu531
**6e**	-9.6		Ile345, Val349, Leu352, Tyr355, Ser353, Leu359, Tyr385, Trp387, Val523, Gly526, Ala527, Ser530, Leu531, Met535, Leu534,
**7**	-9.6	Ser530 (2.76 Å)<br>Leu531 (2.90 Å)	Met113, Val116, Leu117, Arg120, Ile345, Val349, Tyr355, Leu359, Val523, Ala527, Ser530, Leu531
